# Evaluation of a new, rapid, simple test for the detection of influenza virus

**DOI:** 10.1186/s12879-015-0775-5

**Published:** 2015-02-06

**Authors:** Juan Carlos Hurtado, Maria Mar Mosquera, Elisa de Lazzari, Esteban Martínez, Nuria Torner, Ricard Isanta, Patricia de Molina, Tomás Pumarola, Maria Angeles Marcos, Jordi Vila Estape

**Affiliations:** Department of Clinical Microbiology, Hospital Clinic, School of Medicine, University of Barcelona, Barcelona, Spain; Barcelona Centre for International Health Research (CRESIB, Hospital Clinic-Universitat de Barcelona), Barcelona, Spain; Infectious Diseases Unit, Hospital Clinic-IDIBAPS, University of Barcelona, Barcelona, Spain; Public Health Agency of Catalonia, Barcelona, Spain; CIBER Epidemiology and Public Health (CIBERESP), Madrid, Spain; Department of Microbiology, Hospital Universitari Vall d’Hebron (HUVH), Universidad Autónoma de Barcelona (UAB), Vall d’Hebron Institut de Recerca (VHIR), Barcelona, Spain

**Keywords:** Influenza Virus A and B, Isothermal amplification, Real time PCR, Rapid test

## Abstract

**Background:**

Influenza virus infections are responsible for significant morbidity and mortality in both pediatric and adult populations worldwide. Rapid and accurate diagnosis of influenza is necessary for appropriate patient management during the influenza season and for optimal utilization of anti-influenza therapy. We prospectively tested the accuracy of a simple and rapid diagnostic method.

**Methods:**

Ninety-eight samples (nasal and pharyngeal swabs) from patients with upper respiratory tract infection symptoms who presented to primary healthcare centres in Barcelona (Spain) were prospectively analyzed. The samples were collected as part of influenza surveillance program. Samples that had enough volume to make the new test after aliquoting the amount needed to perform routine tests were included. None of the samples were pre-selected as a result of their status in relation to influenza virus. Samples were analyzed by in-house real-time PCR and Alere™ i Influenza A & B (Alere™ i), which uses isothermal amplification of nucleic acids for the qualitative detection of influenza A and B in nasal swabs transported in viral transport media. The two techniques were compared by positive percent agreement (PPA) and negative percent agreement (NPA). Statistical analysis was performed with Stata.

**Results:**

Of the 98 samples analysed 90 were concordant; 46 (46.9%) were positive and 44 (44.9%) were negative. Five samples showed invalid results with the Alere™ i test and could be not re-tested due to insufficient sample volume and were not included in the final statistical analysis. In the 93 remaining samples, the Alere™ i test showed 97% of accuracy having correctly classified 90 samples. We obtained discordant results in 3 samples (3%). The PPA was 93.8% for influenza A and 94.1% for influenza B, and NPA was 100% for influenza A and influenza B virus. In addition, the Alere™ i was very rapid (15 minutes or less) and extremely easy to use.

**Conclusions:**

The Alere™ i test provided a good correlation compared to the real-time PCR test for the diagnosis of influenza. Since this method can be performed in minutes, it allows immediate, accurate clinical decisions to prescribe appropriate antiviral treatment or isolation of patients.

## Background

Influenza virus infections are responsible for significant morbidity and mortality in both pediatric and adult populations worldwide. Rapid and accurate diagnosis of influenza is necessary for appropriate patient management during influenza season and for optimal utilization of anti-influenza therapy [1,2].

Molecular testing, such as real-time PCR, is more sensitive, specific and less time consuming than viral culture and immunofluorescence assays. However, real-time PCR is technically demanding and quite laborious and must be done in a sophisticated laboratory. Rapid antigen-based assays are simple and fast methods that have been widely used for the detection of influenza virus but with its major limitation is their low and widely variable sensitivity (10%–80%) [3-8].

Alere™ i (Scarborough, Maine, USA) is a rapid molecular diagnostic test using nicking endonuclease amplification reaction, a kind of isothermal amplification, and it allows for differential and qualitative detection of influenza virus type A and B from nasal swab specimens in viral transport medium. Alere™ i enables nucleic acid amplification without the need for the long thermal cyclers, allowing result to be obtained in 15 minutes or less. It does not require a DNA purification step which is time-consuming, more complex and costly. For these reasons, it can be performed anywhere, without the need for sophisticated laboratories.

The aim of this study was to compare Alere™ i Influenza A & B with an in-house real-time PCR for influenza virus detection in prospectively recruited patients presenting with flu symptoms during the 2011–2012 influenza season in Barcelona, Spain.

## Methods

### Data and sample collection

From January 7th to April 30th, 2012, excess samples (nasal and pharyngeal swabs) from patients presenting flu-like symptoms at primary care health centers were prospectively analyzed as part of the “Influenza Surveillance in Catalonia, Spain” program available at the Health Department of Generalitat de Catalunya website (http://grip.gencat.cat/ca/la_grip_professionals/documentacio/). This study was approved by the Ethic Committee of Hospital Clinic of Barcelona and patients gave informed consent.

Specimens were collected using swabs and inserted into sample collection tube (kit of 3 ml UTM™ medium with 2 polyester swabs, Copan Diagnostics Inc., Murrieta, CA, USA) and were shipped the same day of collection at a temperature of 2°C to −4°C. Samples that had enough volume to make the new test after aliquoting the amount needed to perform routine tests were included. None of the samples were pre-selected as a result of their status in relation to influenza virus, and were sequentially included in the study.

### Processing sample

**Figure 1:**
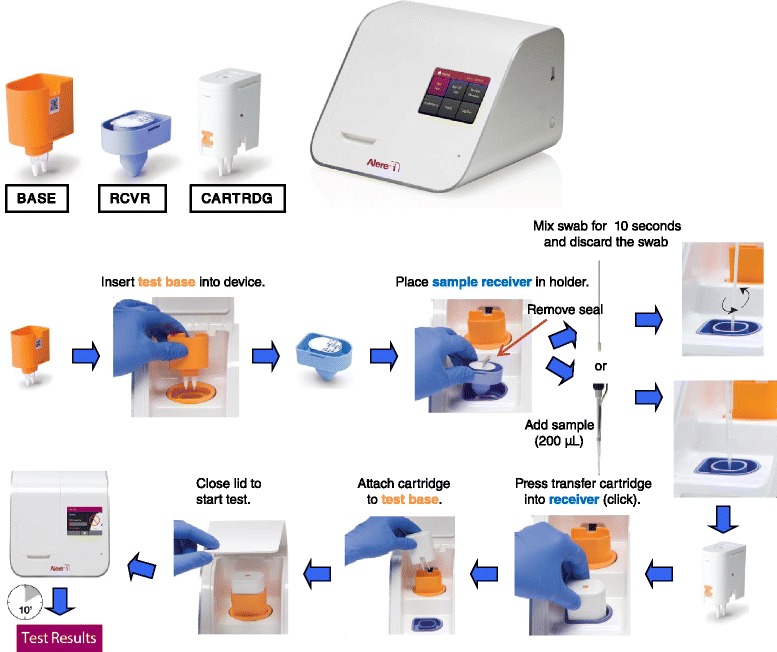
**Steps for run a test with Alere™ i Influenza A&B.** RCVR: Sample receivers. CARTRDG: Transfer Cartridges.

Samples were processed in the laboratory in Biosafety Level 2 Plus facilities, distributed in several aliquots and were processed simultaneously with routine testing within 24 hours of receipt at the laboratory. Samples came weekends were processed within three days. An aliquot with 200 μL of each sample was used to perform the Alere™ i test according to the manufacturer's instructions (Figure 1). Simultaneously, a 300 μL aliquot was taken for total nucleic acids extraction and eluted in 25 μL of RNase-free elution buffer using the automatic QIAsymphony system (Qiagen, Hilden, Germany) according to the manufacturer's instructions. Subsequently, two specific 1-step multiplex real-time PCR was carried out using the Stratagene Mx3000P QPCR Systems (Agilent Technologies, Santa Clara, CA, USA), described elsewhere [9], were used for typing A/B influenza virus (sensitivity was 10 and 10^3^ copies/μL, respectively) and subtyping influenza A virus (sensitivity was 10^2^, 10^3^ and 10 copies/μL for H1, H3 and H5 RNA, respectively) [9].

### Statistical analysis

The Alere™ i test and real-time PCR diagnostic techniques were compared by PPA and NPA, considering the real-time PCR test as an imperfect reference standard. Estimates were reported along their 95% confidence intervals (95% CI) [10-12]. Statistical analysis was performed with Stata (StataCorp. 2013. Stata: Release 13. Statistical Software. College Station, TX: StataCorp LP).

## Results and discussion

Ninety-eight samples were collected from 40 (41%) female and 58 (59%) male patients of whom 46 (47%) were younger than 18 years old. Five samples showed invalid results with the Alere™ i test, of which 3 were positive and 2 negative by PCR. These samples could not be re-tested due to insufficient sample volume and were not included in the final statistical analysis. In the 93 remaining samples, the Alere™ i test showed 97% of accuracy having correctly classified 90 samples; 46 as true positive and 44 as true negative. Among the true positive samples, 30 were identified as influenza A virus and 16 as B virus. The Alere™ i test did not detect virus in any specimens that were not confirmed as positive by the PCR assay. All influenza A virus identified were subtyped by multiplex real-time PCR as influenza A H3. Discrepancies were noted in only 3 samples (2 influenza A virus and 1 influenza B virus), all classified as false negative by Alere™ i. The mean real-time PCR threshold cycle (Ct) values of all 46 positives were 21 while the mean Ct value of the samples with discordant or invalid results was 20.3 and 25, respectively. The PPA of the Alere™ i test was 93.8% for influenza A and 94.1% for influenza B, respectively. The NPA was 100% for both influenza A and influenza B virus (Table 1). The average working time for the Alere™ i test was less than 15 minutes while that for real-time PCR assay was longer than 4 hours.

**Table 1:** **Alere™ i and real-time PCR**

**Organism**	**N° detected: Alere/RT-PCR**				**PPA**		**NPA**	
	**+/+(a)**	**+/−(b)**	**−/+(c )**	**−/−(d)**	**a/(a + c) (%)**	**95% CI**	**d/(b + d) (%)**	**95% CI**
Influenza virus A	30	0	2	44	93,8	79.2 – 99.2	100	92.0 – 100.0
Influenza virus B	16	0	1	44	94,1	71.3 – 99.9	100	92.0 – 100.0

**Alere**™: the Alere™ i Influenza A&B.

**PCR**: in-house real-time PCR.

**PPA**: positive percent agreement.

**NPA**: negative percent agreement.

In the last years, rapid antigen-based assays have been utilized for the detection of influenza virus [13]. Generally, positive results of these rapid methods correlate well with influenza virus infection; however, the major limitation is the low and widely variable sensitivity [14-16]. An ideal rapid influenza diagnostic test should have a diagnostic accuracy approaching or equivalent to the most sensitive methods, such as viral culture or real-time PCR.

In this study, we compared the Alere™ i test with an in-house real-time PCR for the detection of influenza virus. The accuracy of the Alere™ i test was 97% and the overall PPA and NPA were very good with the additional advantage of rapid and easy use. Recently, Bell et al. [17] have also reported good performance on comparing Alere™ i with viral cell culture as the reference method (sensitivity was 93%).

In our study, the mean Ct was 21 (the range was 16–29), it may have been low due to sample collection at the onset of symptoms. Additionally participants were recruited at the time of maximum virus circulation. All patients had a clinical diagnosis of upper respiratory tract infection, which is associated with low Ct. We also included patients less than 18 years, who generally have more viruses in their upper respiratory secretions. The samples studied were collected prospectively during flu season 2011–2012; we believe it is necessary to conduct additional studies to assess the performance of the test against other subtypes of influenza virus.

Our study has some limitations: the percentage of invalid results was proportionally greater than that described by Bell et al. [17]. This fact may be due to the small sample size and some handling errors at the beginning of test use. Other limitation was the impossibility to retest samples with invalid results according to the Package Insert due to the lack of samples. It was of note that the H1N1 subtype was not observed in our study population. Indeed, the H3N2 subtype of influenza virus was found in all the samples, but we did not detect H1N1 or any other subtype, therefore we cannot extend our observations to all isolates. Nevertheless, we think that the test should detect other subtypes including H1N1 such as the data obtained by Nie et al. [18] who found no significant difference on comparing Alere™ i with a PCR technique.

## Conclusions

Alere™ i combines the speed of a rapid antigen with the sensitivity of PCR in the diagnosis of influenza. In addition, it may be used at any time and any day and without specialized personnel. These characteristics make this test particularly valuable for primary care physicians during the influenza epidemic season for point-of-care testing because it allows immediate and accurate clinical decisions to prescribe appropriate antiviral treatment or the isolation of these patients.

## Abbreviations

PCR: Real-time polymerase chain reaction
PPA: Positive percent agreement
NPA: Negative percent agreement
Alere™ i: Alere™ i Influenza A & B
DNA: Deoxyribonucleic acid
Ct: Threshold cycle
