# Erratum: Evaluation of a new, rapid, simple test for the detection of influenza virus

**DOI:** 10.1186/s12879-015-1001-1

**Published:** 2015-09-22

**Authors:** Juan Carlos Hurtado, Mar Mosquera, Elisa de Lazzari, Esteban Martínez, Nuria Torner, Ricard Isanta, Patricia de Molina, Tomás Pumarola, Jordi Vila, Maria Angeles Marcos

**Affiliations:** Department of Clinical Microbiology, Hospital Clinic, School of Medicine, University of Barcelona, Barcelona, Spain; Barcelona Centre for International Health Research (CRESIB, Hospital Clinic-Universitat de Barcelona), Barcelona, Spain; Infectious Diseases Unit, Hospital Clinic-IDIBAPS, University of Barcelona, Barcelona, Spain; Public Health Agency of Catalonia, Barcelona, Spain; CIBER Epidemiology and Public Health (CIBERESP), Madrid, Spain; Department of Microbiology, Hospital Universitari Vall d’Hebron (HUVH), Universidad Autónoma de Barcelona (UAB), Vall d’Hebron Institut de Recerca (VHIR), Barcelona, Spain

Following publication of [1] it has come to our attention that there was an error in the authorship order and in Jordi Vila’s name, which should be Jordi Vila and not Jordi Vila Estape.

We would like to sincerely apologize for the error and any inconvenience caused.
